# Mitochondrial Morphological Features Are Associated with Fission and Fusion Events

**DOI:** 10.1371/journal.pone.0095265

**Published:** 2014-04-14

**Authors:** Laura M. Westrate, Jeffrey A. Drocco, Katie R. Martin, William S. Hlavacek, Jeffrey P. MacKeigan

**Affiliations:** 1 Laboratory of Systems Biology, Van Andel Research Institute, Grand Rapids, Michigan, United States of America; 2 Van Andel Institute Graduate School, Grand Rapids, Michigan, United States of America; 3 Center for Nonlinear Studies, Theoretical Division, Los Alamos National Laboratory, Los Alamos, New Mexico, United States of America; 4 Theoretical Biology and Biophysics Group, Theoretical Division, Los Alamos National Laboratory, Los Alamos, New Mexico, United States of America; 5 Biosciences and Biotechnology Division, Lawrence Livermore National Laboratory, Livermore, California, United States of America; Boston University, United States of America

## Abstract

Mitochondria are dynamic organelles that undergo constant remodeling through the regulation of two opposing processes, mitochondrial fission and fusion. Although several key regulators and physiological stimuli have been identified to control mitochondrial fission and fusion, the role of mitochondrial morphology in the two processes remains to be determined. To address this knowledge gap, we investigated whether morphological features extracted from time-lapse live-cell images of mitochondria could be used to predict mitochondrial fate. That is, we asked if we could predict whether a mitochondrion is likely to participate in a fission or fusion event based on its current shape and local environment. Using live-cell microscopy, image analysis software, and supervised machine learning, we characterized mitochondrial dynamics with single-organelle resolution to identify features of mitochondria that are predictive of fission and fusion events. A random forest (RF) model was trained to correctly classify mitochondria poised for either fission or fusion based on a series of morphological and positional features for each organelle. Of the features we evaluated, mitochondrial perimeter positively correlated with mitochondria about to undergo a fission event. Similarly mitochondrial solidity (compact shape) positively correlated with mitochondria about to undergo a fusion event. Our results indicate that fission and fusion are positively correlated with mitochondrial morphological features; and therefore, mitochondrial fission and fusion may be influenced by the mechanical properties of mitochondrial membranes.

## Introduction

Mitochondria regulate a number of cellular processes including cellular metabolism, proliferation, and apoptosis. Maintenance of mitochondrial homeostasis therefore plays a central role in cellular life-death decisions and is regulated, partly, through the competing processes of mitochondrial fission and fusion. Mitochondrial fission and fusion function to preserve mitochondrial function or eliminate mitochondria beyond repair [1], [2]. A damaged mitochondrion can evade catastrophic failure through fusion with a healthy neighboring mitochondrion. This fusion event allows the mitochondrial population to dilute damage through the mixing of mitochondrial DNA (mtDNA) as well as the exchange of proteins, lipids and small-molecule metabolites [3],[4]. On the other hand, a severely damaged mitochondrion may undergo fission to generate smaller mitochondria that are more easily cleared through a cellular degradation process such as mitophagy [5],[6]. High levels of mitochondrial damage can result in the loss of mitochondrial membrane potential, rendering mitochondria incapable of fusion, a process dependent on inner mitochondrial membrane potential. Consequently, mitochondrial fission can be utilized by the cell to segregate severely damaged mitochondria for degradation [5],[7]. Besides maintaining mitochondrial integrity, coordinated changes in mitochondrial morphology have also been known to play roles in segregating and protecting mtDNA as well as maintaining electrical and biochemical potentials across the double membrane organelle [8],[9].

The execution of several important cellular processes also requires an intricate balance between mitochondrial fission and fusion. Cell division requires mitochondria to fragment to a size that ensures the mitochondria can be segregated properly into the two resulting daughter cells [10],[11]. Recent work by the Lippincott-Schwartz lab revealed a dynamic progression of mitochondrial morphology coordinated with different stages of the cell cycle. In particular, mitochondria were found to form a hyperfused network at the G(1)-S boundary, which provides the cell with increased levels of ATP required for further progression through the cell cycle [10]. Dramatic remodeling of the mitochondrial reticulum is also observed in conjunction with one of the final stages of apoptosis, mitochondrial outer membrane permeabilization (MOMP). A critical step in apoptosis, the release of pro-apoptotic proteins from the inner mitochondrial membrane space through MOMP has been shown to occur simultaneously with extensive fragmentation of mitochondria [6],[12]. Importantly, dysregulation of mitochondrial fission and fusion has been implicated in several diseases, particularly neurodegenerative diseases, and thus underscores the role mitochondrial fission and fusion play in not only maintaining mitochondrial homeostasis, but also in overall cellular viability [3].

The regulation of mitochondrial fission and fusion is controlled by the coordinated action of a series of well-conserved GTPases [2]. The dynamin related GTPase DRP1 is a cytosolic protein that is recruited to mitochondria to drive mitochondrial fission [13]. In mammalian cells, the proteins MFF, MID49 and MID51 recruit DRP1 to mitochondria. Upon recruitment to a mitochondrion, DRP1 forms extended helices around the outer surface of the organelle, which severs the outer and inner mitochondrial membrane [6],[14]. Mitochondrial fusion is mediated by dynamin-related GTPases, MFN1 and MFN2, which are tethered to the outer mitochondrial membrane and function to initiate membrane fusion between neighboring mitochondria through formation of homo- and heteroligomeric complexes [15],[16],[17]. A third GTPase, OPA1, is localized to the inner mitochondrial membrane and facilitates fusion of the inner mitochondrial membrane [16],[18].

Although several factors, including cellular environment, expression and activity of proteins comprising the fission and fusion machinery, are critical in determining mitochondrial fate, it is less clear what role the structural properties of mitochondria play in these dynamics. Because of the physical constraints involved in fission and fusion, we hypothesized that morphological features of mitochondria would be critical determinants of fission and fusion. To test this hypothesis, we combined machine learning with high-resolution kinetic mitochondrial measurements to uncover predictive morphological features of mitochondria contributing to fission and fusion. A random forest (RF) classifier was trained on the basis of 11 morphological and positional features to predict whether mitochondria were more likely to fuse or fragment. Two morphological parameters, mitochondrial perimeter and mitochondrial solidity, were the top two ranked parameters associated with a fission or fusion event, respectively. The identification of morphological parameters predictive of a fission or fusion event demonstrates that mitochondria do undergo architectural changes that are indicative of a future fission or fusion event.

## Results

### Mitochondria are Dynamic Organelles Undergoing Constant Morphological Change

**Figure 1:**
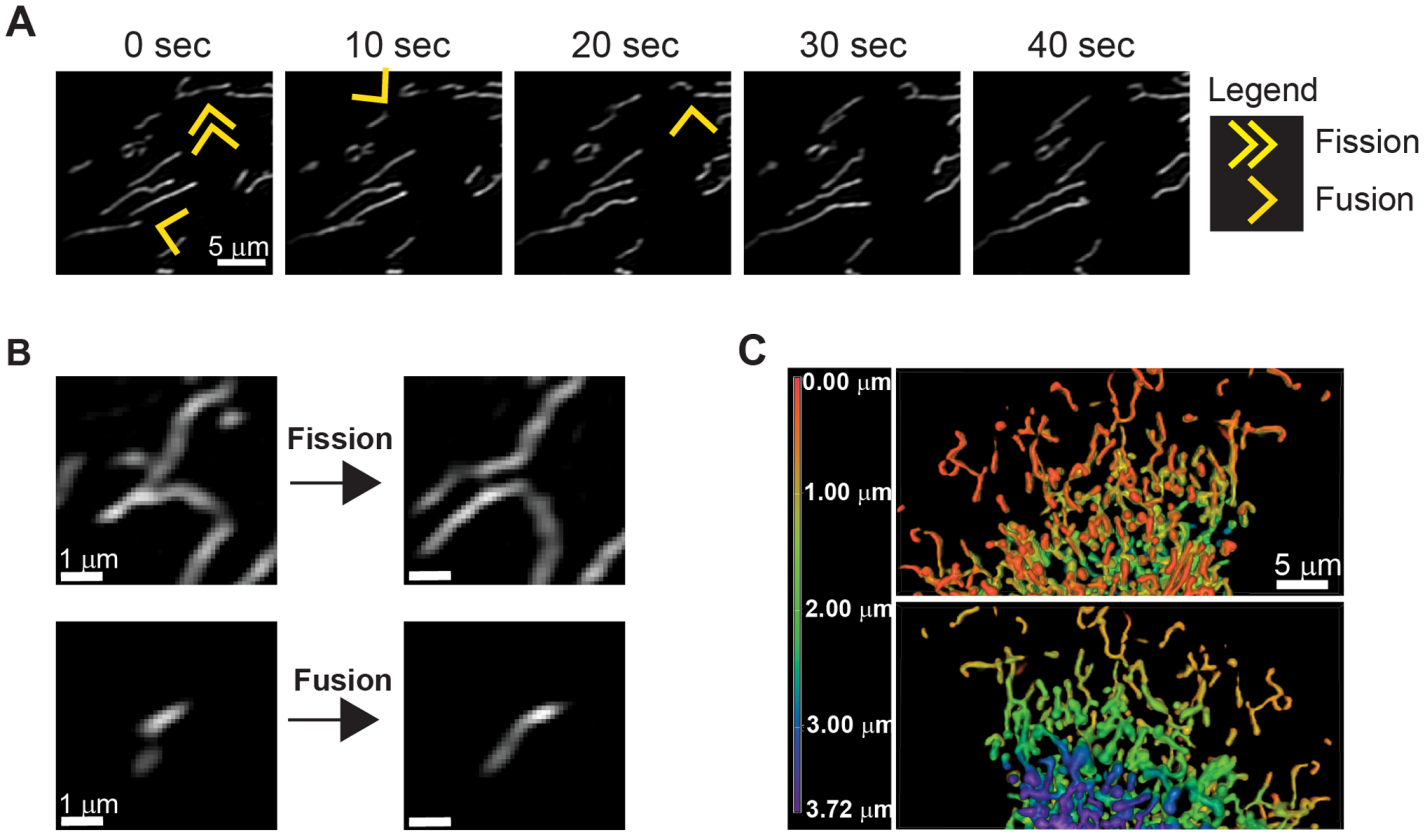
Mitochondrial reticulum undergoes constant morphological remodeling. (A) Time-lapse images of U2OS cells stably expressing mito_EYFP demonstrate fission and fusion events in real time. Double arrows highlight a mitochondrion about to undergo a mitochondrial fission event, while single arrows highlight a mitochondrion about to undergo a mitochondrial fusion event. (B) High magnification (5x) of mitochondrial morphology prior to a mitochondrial fission or fusion event illustrating that heavily branched mitochondria tend to fragment while small, more compact mitochondria tend to fuse. (C) 3D- rendering of mitochondrial z-stack from a U2OS mito_EYFP cell with the mitochondria color coded red to blue to indicate depth of view. The z-stack represents only a portion of the cell with the nucleus oriented beneath the image and the mitochondria extending radially out towards the periphery.

To monitor the dynamics of mitochondrial fission and fusion, we developed a monoclonal U2OS cell line that stably expresses a mitochondrial targeted fluorescent construct (U2OS_mitoEYFP). The construct fuses cytochrome c oxidase subunit VIII (complex IV) to the enhanced yellow fluorescent protein (EYFP) and provides a direct means to visualize mitochondria [19]. We performed mitochondrial co-localization experiments in U2OS_mito_EYFP cells by staining mitochondria with antibodies against endogenous mitochondrial proteins AIF, TOM20, and cytochrome c (Figure S1). Recent evidence has shown that although mitochondrial morphology is altered by various cellular cues (including changing energy requirements or cellular stress), mitochondrial fission and fusion are active under homeostatic conditions and play important roles in the maintenance of mitochondrial populations [1],[2]. Time-lapse fluorescent images of mitochondria within U2OS_mitoEYFP cells revealed that even under homeostatic conditions, fission and fusion events can be observed within a relatively short amount of time (Figure 1A).

To investigate the relationship between the morphological features of mitochondria and mitochondrial fission or fusion, we imaged mitochondria for 5 min, with images taken every 5 s. We examined positional and morphological features of mitochondria just prior to a fission or fusion events by visualizing the organelle in the frame directly preceding the observed event. Mitochondrial morphology varied extensively prior to fission and fusion events; however, we noticed qualitatively that complex mitochondria (longer, branched, and non-uniform in shape) appeared to have a higher propensity to undergo a subsequent mitochondrial fission event. Smaller, spherical mitochondria, on the other hand, were more likely to undergo a future mitochondrial fusion event (Figure 1B). Although the protein availability of the mitochondrial fission and fusion machinery plays an important role in orchestrating the dynamic nature of a particular mitochondrion, we wanted to determine whether the geometric features of mitochondria would play a role in the propensity for mitochondria to fragment or fuse.

### Quantitative Determination of Mitochondrial Fission and Fusion Events

Quantitative measurements of mitochondrial dynamics have been difficult to perform in living eukaryotic cells due to the spatial localization of mitochondria within the cell. Mitochondria tend to cluster in the perinuclear area of the cell and radiate outwards to the periphery. We utilized U2OS cells that are highly amenable to imaging due to these cells having a flat, epithelial morphology. However, despite the relative thinness of U2OS cells, the perinuclear region of the cell is typically 3 to 6 microns in depth which allows several mitochondria to stack on top of each other along the *z*-plane. The thickness at the cell periphery, in comparison, is usually less than 1 micron in depth, minimizing the opportunity for mitochondria to occupy overlapping positions when viewed along the *z*-axis (Figure 1C). Due to the time resolution required to track individual mitochondrial fission and fusion events, we chose to use epifluorescent microscopy to focus on mitochondria at the cell periphery where mitochondrial density is moderate and could be captured in a single snapshot. This method allowed high-confidence for tracking single mitochondria.

To track mitochondrial fission and fusion events in real time, we utilized time-lapse microscopy of individual U2OS_mitoEYFP cells. Briefly, cells grown on a coverglass were cultured in normal growth media and imaged every 5 s for 5 min using an epifluorescent microscope (Nikon TI Eclipse). To capture mitochondrial damage while simultaneously tracking fission and fusion events, we co-stained mitochondria with Mitotracker Red CMXRos. This red fluorescent dye localizes to mitochondria and its signal intensity is dependent on mitochondrial membrane potential. Tracking membrane potential changes throughout the time series revealed that mitochondrial membrane potential was maintained throughout the time series. In a few isolated mitochondria, we could observe loss of Mitotracker which indicates a loss in mitochondrial membrane potential (Figure S2). In these situations, loss of membrane potential led to future fission events, consistent with previously published results that have found that mitochondrial fusion is dependent on mitochondrial membrane potential [5],[7].

**Figure 2:**
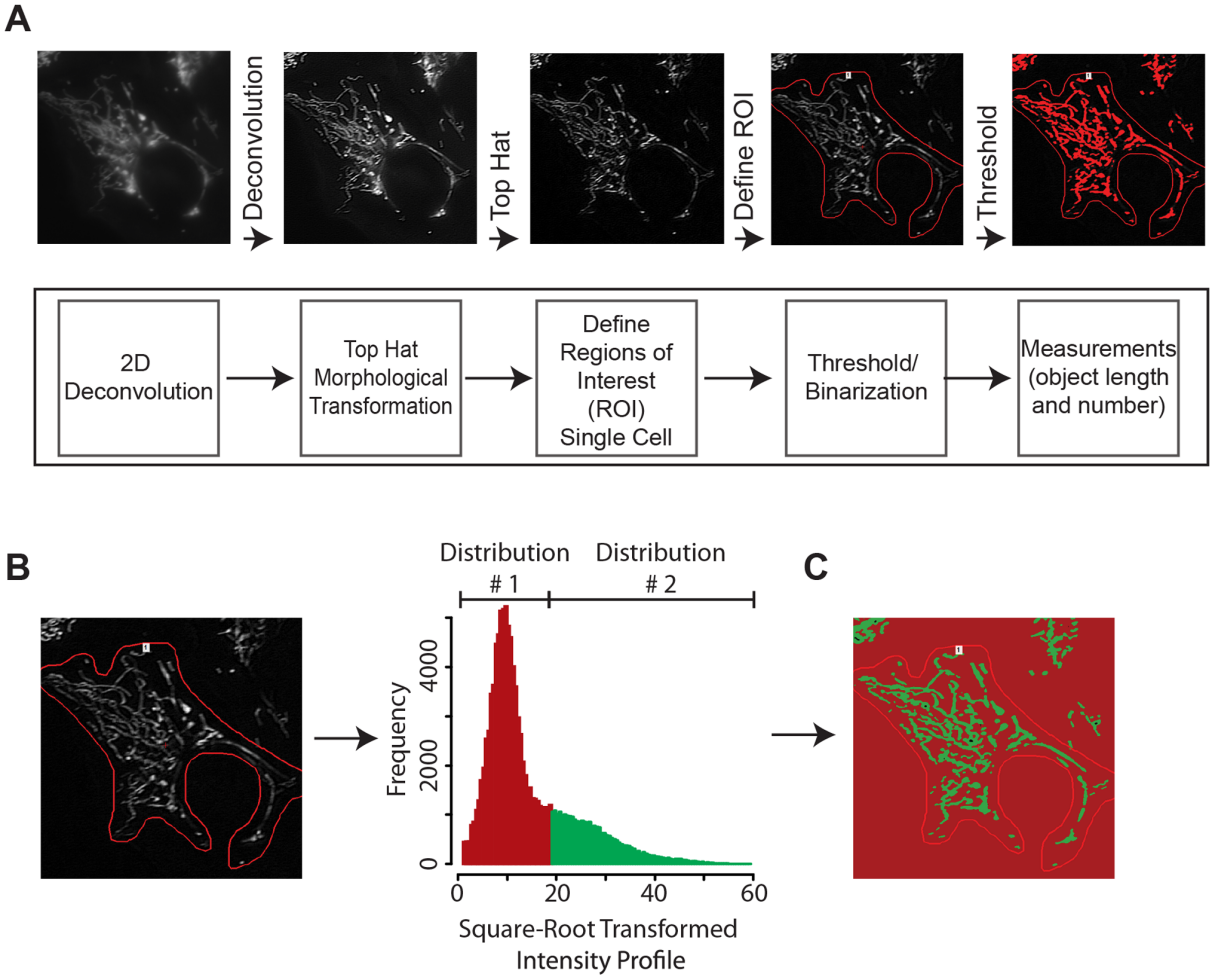
Quantification design for tracking mitochondrial morphology dynamics using mito_EYFP. (A) Quantification schematic used to process images for analysis. Mitochondria were imaged in U2OS_mitoEYFP cells by fluorescent microscopy and were subjected to image processing that involved 2D deconvolution, top hat morphological transformation, intensity thresholding, and object quantification. (B) Intensity profiles from each individual cell were used to determine the thresholding boundaries for each image. Intensity profiles depict single distribution with a prominent right-hand elbow that we separated and termed distribution 1 and 2: pixels within distribution 1 are highlighted red and represent non-mitochondrial pixels whereas pixels within distribution 2 are highlighted green and represent mitochondrial pixels. (C) Split color of mitochondrial image in 1B highlighting location of pixels within each distribution. Mitochondrial measurements were performed with a threshold that excluded pixels in distribution 1.

**Figure 3:**
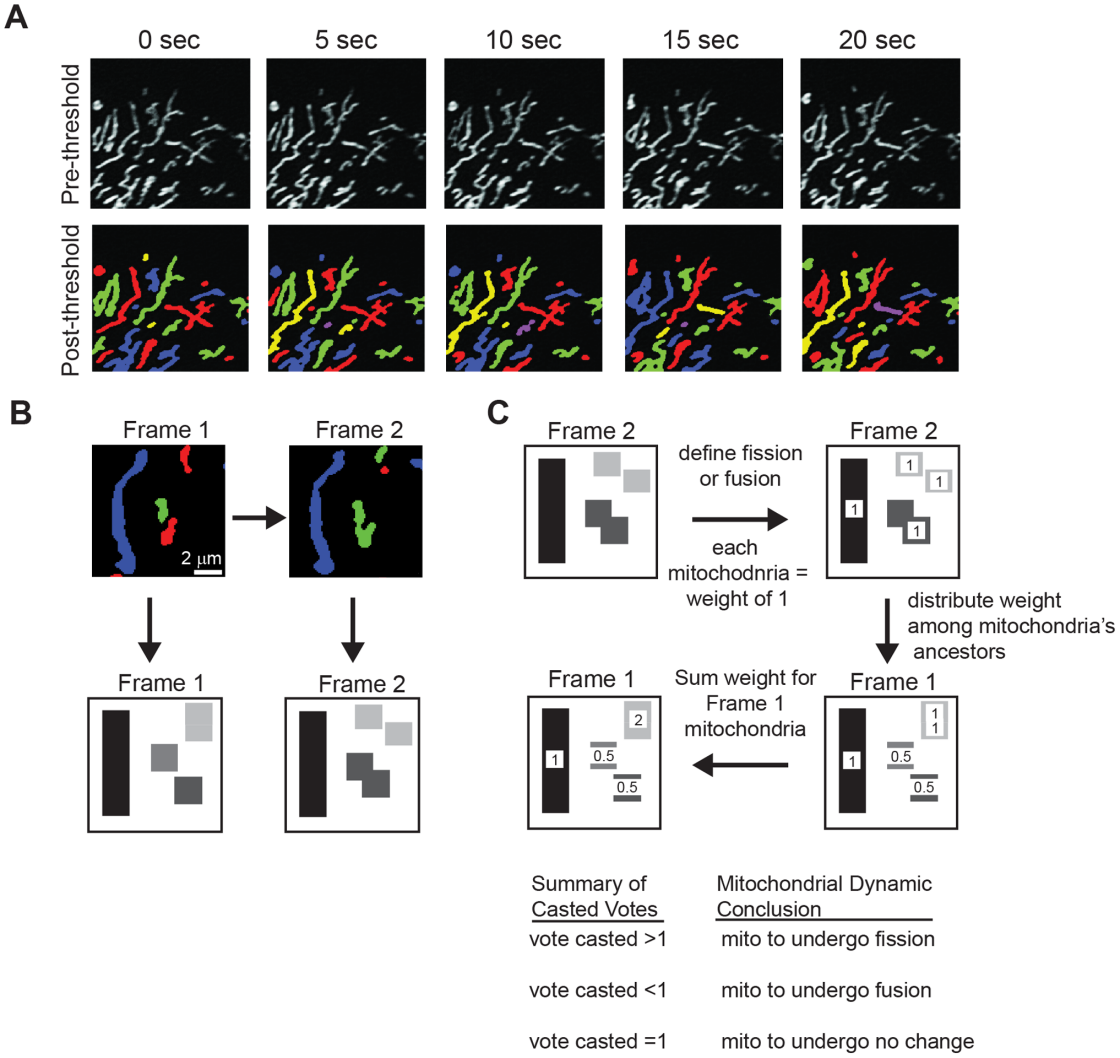
Quantitative characterization of mitochondrial fission and fusion events. (A) Thresholded images following the schematic described in Figure 2 segregates individual mitochondria to be tracked for identification of fission or fusion events. (B) Mitochondria in Frame 1 and 2 were tracked as discrete regions through time to monitor for fission and fusion events. (C) Simplified diagram illustrating quantitative characterization of a fission and fusion event occurring amongst mitochondria as they progress from one frame to the next (Frame 1 to Frame 2). Mitochondria in Frame 1 with a score of less than 1 will undergo a mitochondrial fusion event in Frame 2. Mitochondria in Frame 1 with a score higher than 1 are marked as mitochondria that will undergo a mitochondrial fission even tin Frame 2. Mitochondria with a score of 1 will undergo neither a fission or fusion event in Frame 2.

Identification of individual fission and fusion events was achieved following a detailed quantification protocol that incorporated analysis software as described in Material and Methods (*see Image Processing and Mitochondrial Quantification*). This quantification protocol yielded a numerical summary describing several mitochondrial features in single cells (Figure 2A). Single cells were defined by regions of interest (ROIs), and recognition of mitochondria was determined by thresholding the image based on the intensity profile of each ROI. We used intensity thresholding to accurately distinguish true mitochondria pixels from background fluorescence (Figure 2B, C). Overall, this image thresholding and binarization protocol allowed us to standardize and automate the selection of mitochondrial objects, which were manually inspected and compared to original images before being exported to MATLAB for analysis (Figure 3A).

### Identification of Mitochondrial Fission and Fusion Events

Next, we used computational modeling and analysis to provide an unbiased mechanism to detect fission and fusion events. To confirm that the computational model accurately identified fission and fusion events, individual frames were manually inspected. A mitochondrion poised to undergo a fission or fusion event was defined as a mitochondrion that would undergo an event in the subsequent frame, or in 5 s time. For each frame within a time series, a reference frame (Frame 1, time *t* = 0) was chosen and compared with the subsequent image (Frame 2, time *t* = 5 s) (Figure 3B). Each mitochondrial object was defined as a distinct region and the regions were tracked through time as described in Material and Methods (see *Determination of Fission and Fusion Events*). Mitochondrial fission was defined as an event where a mitochondrion divided into at least two mitochondria. Mitochondrial fusion was defined as an event where at least two mitochondria joined to form a new mitochondrion. If neither of these events occurred for the mitochondrion in question, it was defined as having performed neither and its geometric parameters were not considered further.

Computationally, a mitochondrion was determined to have either undergone (1) a fission event, (2) a fusion event, or (3) no event, based on the relationship between mitochondria in two sequential frames, which for purposes of discussion, we will refer to as Frames 1 and 2 (Figure 3B). The fate of the mitochondria from Frame 1 to Frame 2 was determined by considering each mitochondrion in Frame 2 as a single unit with a uniform “weight” of 1 (regardless of size). The weight of each mitochondrial unit was then distributed among its ancestors in Frame 1 and used to define what type of event that mitochondrion would undergo before the subsequent frame. The ancestor(s) of each mitochondrion were determined as described in Material and Methods (see *Determination of Fission and Fusion Events*). For example, a mitochondrion that undergoes a fusion event with an adjacent mitochondrion will receive a fraction of the “weight” from the newly fused mitochondria in Frame 2, and a mitochondrion that will fragment will sum the distributed weights from its newly fragmented mitochondria in Frame 2. In this way, a mitochondrion in Frame 1 with a score higher than 1 was recorded as poised to undergo fission, a score lower than 1 was recorded as poised to undergo fusion, and a score equal to 1 was recorded as neither (Figure 3C). We tracked the fission and fusion potential of mitochondria throughout the entire time series (5 s intervals for 5 min, 60 total frames). Our computational algorithm identified a total of 559 mitochondria that underwent a fusion event and 237 mitochondria that underwent a fission event.

As in earlier work in which fission and fusion events were tracked through labeling of the entire mitochondrial reticulum [20],[21]. Our use of the mito_EYFP fluorescent construct allowed us to mark all of the mitochondria within a cell, and track the dynamics of a mitochondrial population within a common cell over time. Here, we used this imaging technique to study the dynamics of individual mitochondria, taking into account local mitochondrial density. One concern with this approach is that identification of fission and fusion events may be confounded by the high motility of mitochondria. In other words, there is the potential for two mitochondria simply passing each other to be confused as a sequence of fission and fusion events. To minimize false detection of fission and fusion events, we only scored events that persisted for at least two frames within a time series (i.e., 10 s). Thus, a fission (or fusion) event was only scored as such if the relevant mitochondrial object(s) maintained a fragmented (or fused) state for at least 10 s after the putative event.

To provide experimental mitochondrial fusion validation, we used photoactivatable GFP (PA-GFP) in the context a mitochondrial dye that stained the entire mitochondrial population. Briefly, a polyclonal population of cells expressing PA-GFP (green) was costained with Mitotracker (red), photoactivated, and imaged for 5 min (Figure S3). Here, a PA-GFP identified fusion event was marked by the co-merging of red and green fluorescence to generate a fused yellow mitochondrion. Due to the nature of the assay and the requirement of local photoactivation, our identification of fusion events using a photoactivatable approach provided results on local fusion events, thus making PA-GFP ineffective in quantifying global fission and fusion events in an entire cell. Subsequently, we focused on identifying fission and fusion events that utilized a mitochondrial labeling system (mito_EYFP) that takes into account fission, fusion, and the entire mitochondrial population.

### Perimeter and Solidity are Predictive Features of Mitochondrial Fission and Fusion

Having completely identified fission and fusion events in the dataset, we next sought to determine if the morphological or positional properties of mitochondria influenced fission and fusion events. An ensemble learning algorithm was used to develop a classifier capable of distinguishing mitochondria poised to undergo fission from mitochondria poised to undergo fusion. Several morphological and positional features were computed for each mitochondrion just prior to the identified fission or fusion event (Table 1). These parameters were then used to train a random forest (RF) classifier to predict whether a mitochondrion is more likely to fuse or fragment. The RF consists of a collection of decision trees that use predictable inputs, here, the mitochondrial parameters, to vote for a particular output, mitochondrial fission or fusion.

**Table 1:** Morphological and Positional Features.

Feature	Definition
Solidity	The fraction of pixels in the smallest convex polygon (completely containing the mitochondria) that are also mitochondrial pixels
Perimeter	Sum of the distance between adjacent pixels around the border of the region (in microns)
Number of necks	Number of branch points in a mitochondria
Area	Two dimensional sum of pixels in the mitochondria multiplied by the area of each pixel (in microns)
Nearest neighbor distance	Distance (in microns) between the mitochondria and its nearest neighboring mitochondria
Extent	The fraction of pixels in the smallest rectangle (completely containing the mitochondria) that are also mitochondrial pixels
Width of narrowest neck	Width of the smallest neck/branch point on a mitochondria (measured in micron)
Eccentricity	A measure of deviation from circular shape
Orientation relative to nucleus	Direction of major axis of the mitochondrion relative to the position of the nucleus in the cell
Euler number	A topological measure that indicates the number of holes in the object

Description of the parameters queried for their potential to predict fission and fusion events. There morphological and positional features were used as inputs in the random forest algorithm used to identify which features were predictive of a subsequent fission or fusion event.

Development and analysis of the RF model generated a ranking for the importance of 11 features, which are listed in Table 1. Ranking was determined by calculating the increase in out-of-bag (OOB) prediction error when each feature was removed from consideration (Table 2). The OOB error provides a measure of how well the model generalizes to unseen data. We found that two features, perimeter and solidity, were the best predictors of whether a mitochondrion would fragment or fuse (Table 2).

**Table 2:** Feature Ranking of Control Cells.

Rank	Features	All events	Fission events	Fusion events
1	Perimeter	0.059±0.010	0.087±0.009	0.047±0.009
2	Solidity	0.052±0.009	0.099±0.010	0.032±0.
3	Number of necks	0.037±0.007	0.055±0.007	0.029±0.007
4	Area	0.035±0.006	0.067±0.008	0.022±0.005
5	Nearest neighbor distance	0.026±0.004	0.052±0.005	0.015±0.003
6	Extent	0.020±0.005	0.026±0.005	0.017±0.005
7	Neighbor surface within 10 µm	0.018±0.003	0.035±0.004	0.011±0.002
8	Width of narrowest neck	0.015±0.003	0.039±0.004	0.005±0.002
9	Eccentricity	0.005±0.002	–0.004±0.002	0.009±0.002
10	Euler number	0.003±0.002	–0.0001±0.001	0.004±0.002
11	Orientation relative to nucleus	0.002±0.002	–0.001±0.002	0.004±0.002

Mitochondrial Features from Table 1 were ranked in order of importance to the prediction algorithm used to predict a future fission or fusion event. Standard deviation indicates uncertainty. Each parameter’s score of importance was calculated considering only its ability to predict both fission and fusion events (“All events”), only fusion events and only fission events.

**Figure 4:**
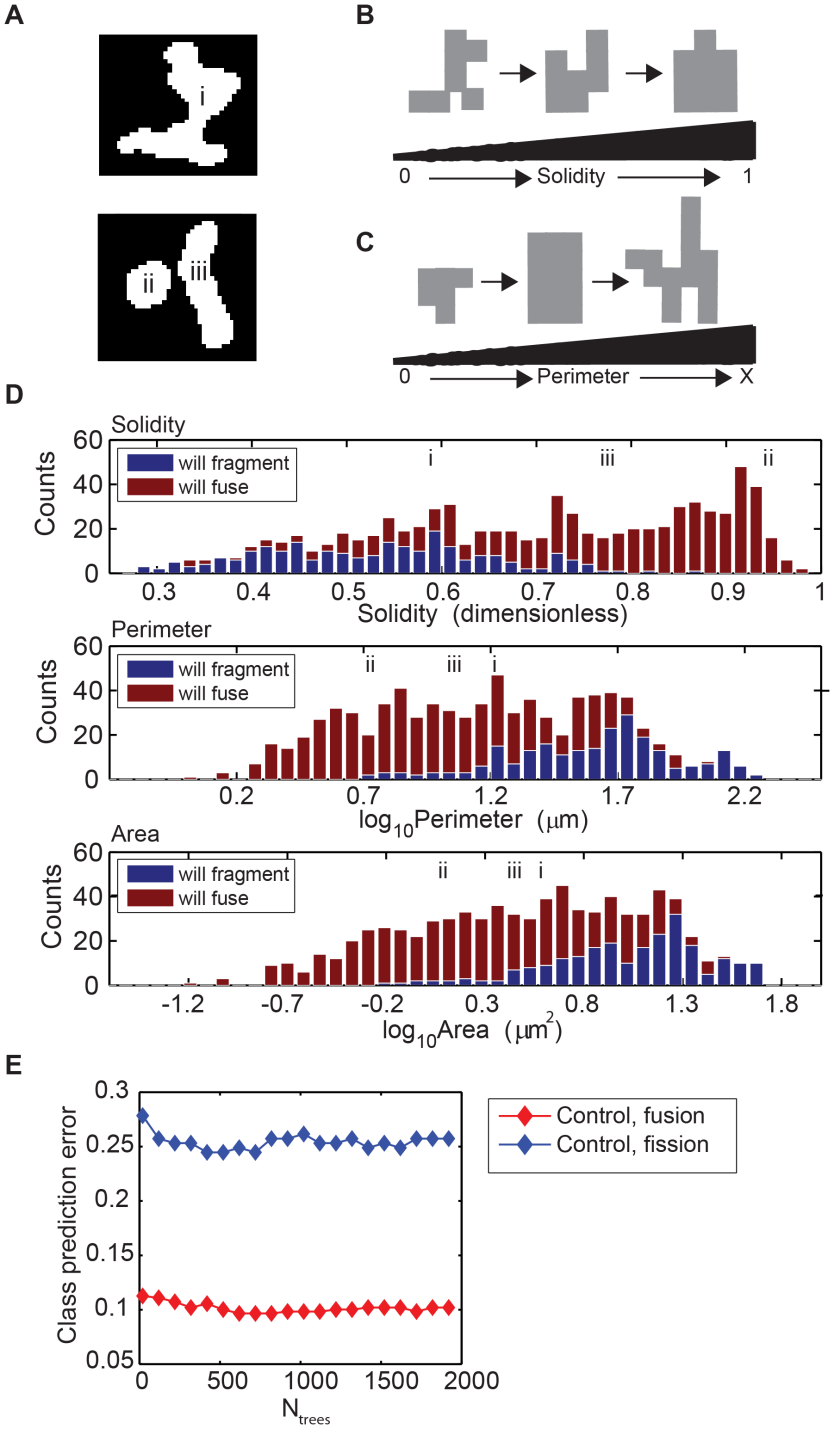
Mitochondrial features, solidity, perimeter, and area, segregate mitochondria classified to fragment or fuse. (A) Thresholded mitochondria annotated i–iii represent three examples of mitochondria identified to undergo a fission or fusion event in the subsequent frame and will be used in panel B to provide context for the shape and size of the mitochondria at different stages along the x-axis (see i–iii notation in population distributions for solidity, perimeter, and area). (B) Mitochondrial solidity is defined as the fraction of pixels contained with a convex polygon (fitted around a mitochondrion) that is also mitochondrial pixels. Low solidity (close to 0) tends to describe highly tortuous mitochondria that are not uniform in shape while high solidity values (closer to 1) tends to describe mitochondria that are more uniform in shape and do not contain a high level of branching. (C) Mitochondrial perimeter is defined as the number of exterior mitochondrial pixels multiplied by the length of the pixels, in microns. While perimeter and area of mitochondria are highly correlated, mitochondria of similar area can have varied perimeters depending on the level of branching and morphology complexity. (D) Feature distributions of solidity, perimeter, and area. The red and blue histograms characterize the distribution of solidity (top), perimeter (middle), and area (bottom) across the population of mitochondria poised to undergo fusion or fission, respectively. Values are binned as indicated on each axis and the number of mitochondria mapping to each bin is indicated on each y axis. (E) Class prediction errors for the random forests calculated for fusion events (red) and fission events (blue). Error decreases as the size of the forest exceeds ∼100 trees and reaches a minimum shortly thereafter.

Ranking the importance of the 11 features for prediction of fission events only (or fusion events only), perimeter and solidity still remained the two best predictors. Perimeter, measured as the number of pixels forming the boundary of a mitochondrial region, and solidity, a measure of shape complexity (higher solidity indicates a more compact shape), are geometric parameters descriptive of the shape of the mitochondria. A mitochondrion therefore, that is irregular in shape (e.g., branched and/or tortuous) tends to have a higher perimeter and a lower solidity than a mitochondrion that is compact (e.g., round or oval) (Figure 4 A–D).

Solidity was positively correlated with the likelihood of a future fusion event, whereas perimeter was positively correlated with the likelihood of a future fission event (Figure 4D). This finding is consistent with our observation noted earlier that large, tortuous mitochondria (i.e. high perimeter) tend to fragment (Figure 1B). Like perimeter, the “area” of a mitochondrion and the number of its “necks” were positively correlated with a propensity to fragment. The number of necks is a morphological parameter that we introduced to measure shape complexity: it corresponds to the number of branch intersections. In addition to number of necks, we also evaluated the minimum neck width as a morphological feature. Surprisingly, minimum neck width was not particularly informative, suggesting that the number of branches seems to have a greater effect on mitochondrial fission/fusion dynamics than their widths.

The least distance to the nearest mitochondrial neighbor, as well as the length of the neighboring surface within 10 µm, are both positional parameters that reflect the relative density of mitochondria in the local neighborhood of a mitochondrion. Both positional parameters were positively correlated with the likelihood of fusion, supporting the mechanism that mitochondrial fusion must first be initiated by developing interactions between neighboring mitochondria. Several features including extent, eccentricity, Euler number, and orientation relative to the nucleus (radial alignment of the principal axis) showed little or no predictive value compared to the features already discussed.

Including all features, the RF model achieved approximately 86% accuracy, or a 14% OOB error rate (which is the final misclassification probability calculated during training of the RF model) in discriminating mitochondria that will fragment or fuse. The OOB error rate is insensitive to over fitting, and will usually overestimate the true error rate of the forest applied to the new data [22]. The 14% error rate is the weighted mean of the class error rates for identifying mitochondria that will fragment or fuse. Interestingly, the algorithm performed significantly better in predicting a subsequent fusion event as opposed to a fission event (Figure 4E). We attribute this performance feature of the RF model to the inability of sufficiently small mitochondria to further divide, making the prediction that they will fuse with a neighbor rather than fragment almost certain.

The populations of mitochondria poised for fission and fusion have overlapping but distinct distributions of feature values. In Figure 4 we show the distributions of solidity, perimeter, and area for mitochondria poised for fission or fusion. Consistent with the previous observations regarding prediction error, large and tortuous mitochondria have a higher propensity to undergo a fission event rather than a fusion event. Likewise, small, round mitochondria with solidity of ≥0.8 or perimeter ≤100 µm (log_10_ = 2) have a higher propensity to participate in mitochondrial fusion events (Figure 4D).

Given our findings that perimeter and solidity are associated with future fission and fusion events, we wanted to investigate whether mitochondria undergo dynamic morphological changes in preparation for these events. To do this, we tracked mitochondrial solidity and perimeter in the time period leading up to a series of 1-to-2 fission or 2-to-1 fusion events. To track changes in mitochondrial perimeter and solidity (perimeter –P, solidity –S), these values were normalized to the profile of the mitochondria just prior the identified events (P_0_ and S_0_) and plotted as P/P_0_ and S/S_0_ over time (Figure S4). Traces shown in Figure S4 represent the mean change in perimeter or solidity for mitochondria identified to undergo a fission event (blue) or fusion event (red). The shaded areas about the trace represent standard error. These results indicate that while mitochondrial morphology remains relatively unchanged prior to an identified fusion event, there are observable changes for mitochondria prior to an identified fission event. Specifically, we found that mitochondria demonstrated increases in perimeter and decreases in solidity prior to the fission events. This is consistent with our finding that perimeter and solidity are positively and negatively correlated with subsequent fission events, respectively. The observation that mitochondrial morphology was dynamically altered leading up to a fission event, but not a fusion event, may reflect known morphological changes required in preparation for mitochondrial fission compared to fusion. Mitochondrial fusion, which is initiated by the tethering of OMM localized GTPases, MFN1 and MFN2 likely does not require dramatic alterations in mitochondrial morphology prior to the event [15],[16],[17]. In contrast, several studies have demonstrated significant conformal changes in mitochondrial morphology in preparation for a mitochondrial fission event [23],[24]. Specifically constriction of the organelle driven by the endoplasmic reticulum or the molecular fission machinery is a critical step to occur prior to mitochondrial scission and may represent the fluctuations in mitochondrial morphology observed just prior to the identified fission event.

### Morphological Characterization of Mitochondria from Fission Mutants Compared to Control

**Figure 5:**
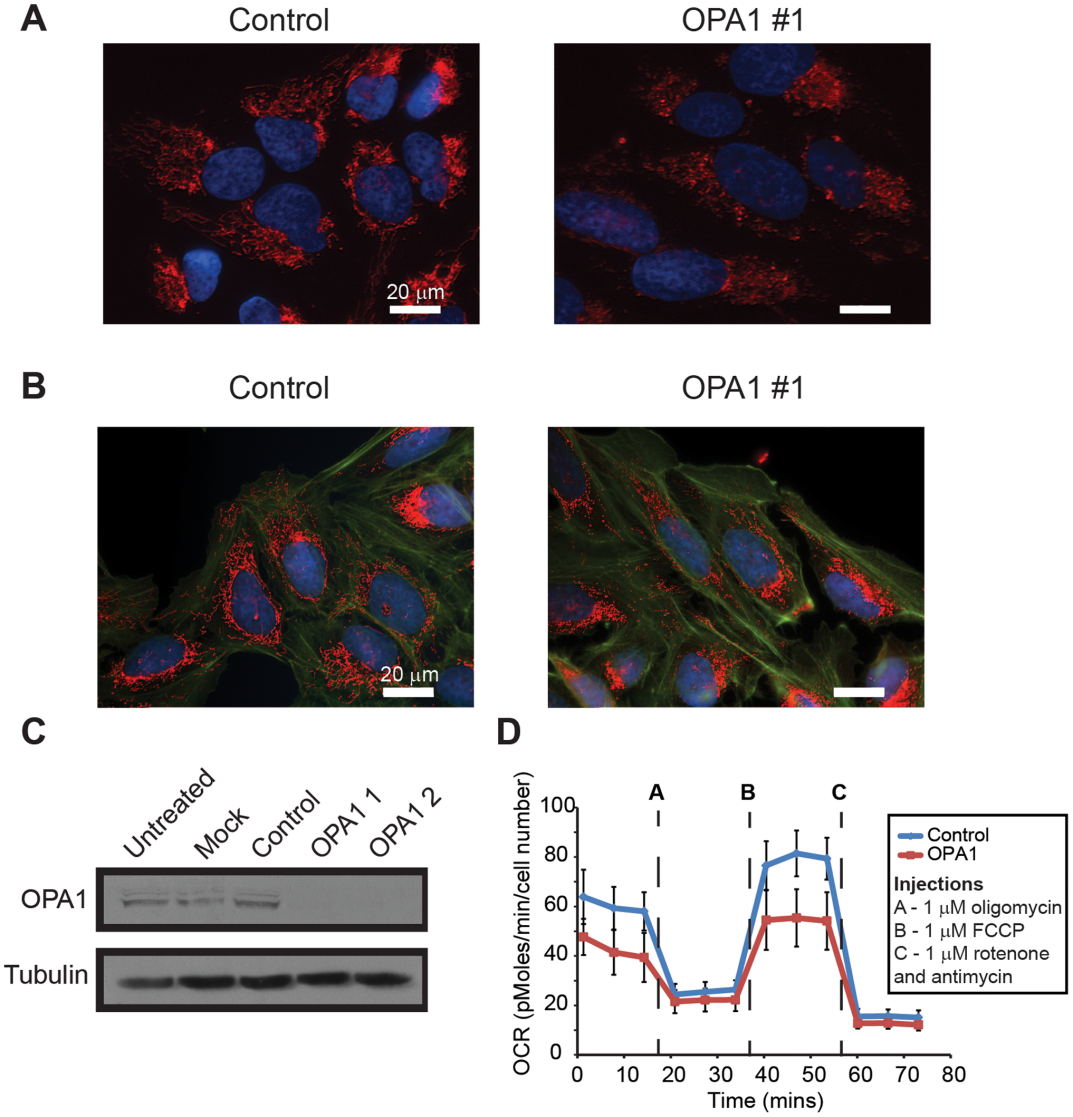
Characterization of mitochondrial health and function. (A) Immunofluorescent images of U2OS cells following targeted knockdown of OPA1 compared to control for 48 hours. Cells were labeled with MitoTracker Red CMXros (mitochondria, red), Phalloidin (actin, green), and Hoechst (nuclei, blue). (B) Immunofluorescent images of U2OS cells labeling endogenous cytochrome c (red) and nuclei (blue, Hoechst) following 48 hours of knockdown for OPA1 and control. (C) Knockdown was confirmed by probing for endogenous OPA1 protein levels via western blot (Untreated – lysates from untreated cells, Mock – lysates from cells transfected with lipid only, Control – lysates from cells transfected with control nonspecific siRNA, OPA 1 and 2– lysates from cells transfected with two different siRNA targeted against OPA1). (D) Respiratory potential of OPA1 knockdown cells (48 hrs) compared to control using the Seahorse metabolic analyzer. Real time measurements of oxygen consumption rate (OCR) were obtained basally and then after treatment with oligomycin (ATP synthase inhibitor), FCCP (ETC accelerator), and Rotenone+Antimycin (ETC inhibitors).

To determine whether mitochondrial perimeter and solidity would still associate with fission and fusion events when the mitochondrial architecture was dramatically altered from the normal state, we treated U2OS_mitoEYFP cells with siRNA targeted against the mitochondrial fusion regulator, *OPA1*, which resulted in highly fragmented mitochondria (Figure 5). Mitochondrial fission and fusion events can be affected by several factors including mitochondrial health, mitochondrial damage and various forms of cellular stress. Mitochondrial morphology changes arising from non-homeostatic conditions could affect the interpretation of how the shapes and spatial distribution of mitochondria are associated with a fission or fusion event. Therefore, we experimentally assessed the condition of mitochondria under our experimental conditions to ensure the mitochondria were not subject to confounding factors that could influence overall mitochondrial morphology. In control and *OPA1* knockdown cells (Figure 5C), mitochondrial localization of cytochrome c was maintained, indicating that the mitochondrial membranes were intact with little to no membrane permeabilization (Figure 5A). Cytochrome c is localized to the inner mitochondrial membrane space and associates with the inner mitochondrial membrane under homeostatic conditions, but is released into the cytosol following the induction of apoptosis. Cytochrome c localized to the mitochondria confirms that the mitochondria were healthy at the start of the time lapsed imaging and this maintained throughout the time series. In addition, we observed similar localization of Mitotracker to the mitochondria in both control and *OPA1* knockdown cells (Figure 5B). Mitochondrial dysfunction is often marked by a loss of membrane potential, and the similarity in Mitotracker labeling of the mitochondria observed for control and *OPA1* knockdown provided additional evidence that the mitochondria imaged in our experiments were healthy.

To further characterize mitochondrial respiratory potential in both control and *OPA1* knockdown cells, we used the Seahorse metabolic analyzer to measure oxygen consumption rate (OCR), and thereby obtain an assessment of mitochondrial respiration (Figure 5D). Real time measurements of OCR were collected following treatment with the ATP Coupler oligomycin (Injection A), which inhibits ATP synthesis, the ETC accelerator FCCP (Injection B), which promotes maximal respiration, and two mitochondrial electron transport chain inhibitors antimycin A and rotenone (Injection C), which essentially disrupt the proton gradient and inhibits ATP generation. In both control and *OPA1* knockdown, the mitochondria were respiratory competent, although *OPA1* knockdown cells were found to exhibit a decrease in respiratory potential compared to control as reported previously (Figure 5D) [23],[24]. Overall, the results reported above support the idea that the morphological features used in machine learning reflect properties of healthy mitochondria under homeostatic conditions, as opposed to features of damaged mitochondria coping with stress.

**Figure 6:**
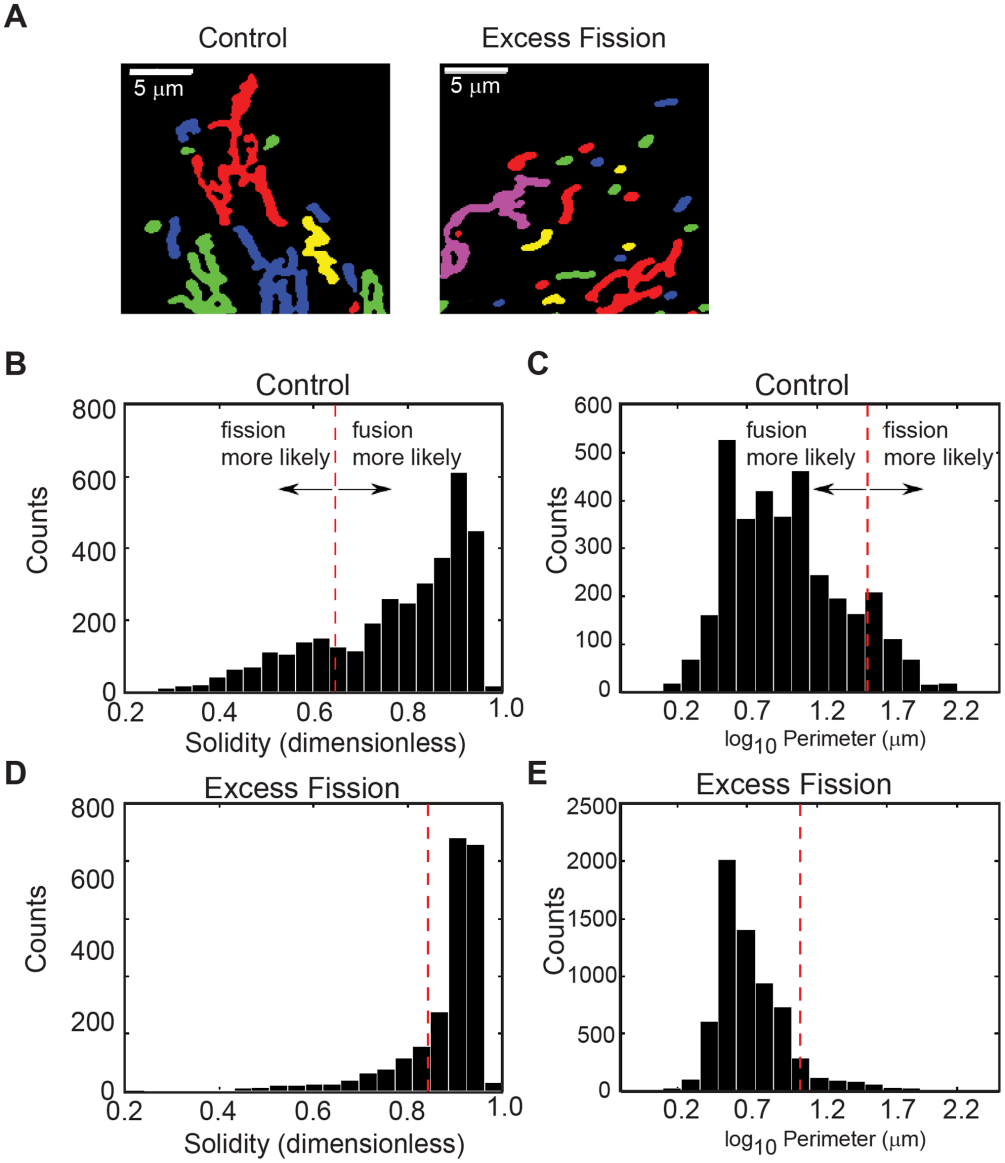
Morphological characterization of mitochondria from fission mutants. (A) Thresholded mitochondria in U2OS_mitoEYFP cells following 48 hour knockdown with control or OPA1 specific siRNA. (B–E) Probability distribution of solidity and perimeter measurements for all mitochondria despite whether they will fragment or fuse in control cells (B and C, respectively) and OPA1 knockdown cells (D and E, respectively). Dashed red lines indicate decision boundary computed from a single classification tree fixed to one bifurcation, trained only on the data from fragmenting/fusing mitochondria in the feature in question.

Time-lapse images of *OPA1* knockdown cells revealed that compared to control cells, the mitochondria were fragmented, as expected, due to the loss of *OPA1* (Figure 6A). Identification of mitochondrial fission and fusion events was again performed as described in Figure 3, and the morphological and positional features of the mitochondria were used to train a RF built solely on the data from the *OPA1* knockdown cells. Development and analysis of the RF model once again generated a ranking for the importance of the 11 features considering the morphological and positional features from OPA1 knockdown cells alone (Table 3). Remarkably, the order and significance of the different features is similar to that obtained for control cells, suggesting that the mechanisms controlling mitochondrial morphological features prior to a fission or fusion event are conserved even in instances when the morphology has been dramatically altered.

**Table 3:** Feature Ranking of OPA1 Knockdown Cells.

Rank	Features	All Events	Fission Events	Fusion Events
1	Solidity	0.076±0.014	0.102±0.012	0.064±0.012
2	Perimeter	0.069±0.013	0.089±0.010	0.058±0.012
3	Area	0.050±0.010	0.080±0.009	0.034±0.008
4	Nearest neighbor distance	0.033±0.006	0.060±0.006	0.021±0.004
5	Neighbor surface within 10 µm	0.033±0.006	0.053±0.006	0.024±0.004
6	Number of necks	0.032±0.007	0.073±0.009	0.012±0.005
7	Extent	0.028±0.007	0.023±0.006	0.029±0.007
8	Width of narrowest neck	0.021±0.004	0.051±0.006	0.006±0.003
9	Eccentricity	0.007±0.003	−0.008±0.002	0.015±0.003
10	Euler number	0.002±0.001	0.003±0.001	0.001±0.001
11	Orientation relative to nucleus	0.001±0.002	−0.001±0.002	0.003±0.001

Mitochondrial Features from Table 1 were ranked in order of importance to the prediction algorithm used to predict a future fission or fusion event in OPA1 knockdown cells. Standard deviation indicates uncertainty. Each parameter’s score of importance was calculated considering only its ability to predict both fission and fusion events (“All events”), only fusion events and only fission events.

Given the alteration in mitochondrial morphology in *OPA1* knockdown cells compared to control, we examined whether there was a shift in the threshold (considering perimeter and solidity parameters) at which fission takes place. Population distributions of perimeter and solidity values for all observed mitochondria, regardless of their proximity to a fission or fusion event, were plotted and used to demonstrate the decision boundary for a subsequent fission and fusion event computed from a single classification tree (Figure 6 B–E). Considering solidity as the only predictor for a subsequent fission or fusion event, mitochondria from control cells with a solidity measurement larger than 0.65 were predicted to have a higher likelihood to fuse with a neighboring mitochondrion, whereas mitochondria with a solidity measurement less than 0.65 were predicted to have a higher likelihood to undergo a fission event (Figure 6B). Similarly, mitochondria from control cells with a perimeter larger than 730 µm (log_10_ = 2.9) have a higher propensity to undergo a fission event and mitochondria with a small perimeter have a higher propensity to fuse with neighboring mitochondria (Figure 6C). For comparison, the decision boundary for fission and fusion likelihood, considering solidity and perimeter for mitochondria from *OPA1* knockdown cells was 0.84 and 240 µm (log_10_ = 2.4), respectively (Figure 6D–E). Decision boundaries for each variable indicate that fission occurs at higher threshold values of solidity and smaller threshold values of perimeter in *OPA1* knockdown cells compared to control.

## Discussion

Mitochondrial morphology is controlled by two highly conserved processes, mitochondrial fission and fusion. Mitochondrial fate is influenced by several factors including cellular environment, metabolic demand, and the stoichiometry of fission and fusion regulators [1],[2],[16]. Interestingly, the findings from this report suggest that the morphology of mitochondria may play a role in impacting their fates. The two factors that were found to be the most predictive of a fission or fusion event, perimeter and solidity, emphasize the finding that mitochondria poised for a fission or fusion event have different morphological features.

The importance of mitochondrial shape in predicting a subsequent fission or fusion event may highlight the role of mitochondrial membrane organization in regulating fission or fusion events. OPA1 has been known to regulate the curvature and complexity of the inner mitochondrial membrane, supporting a role for mitochondrial membrane structure in the regulation of mitochondrial fusion [25]. Similarly, mitochondrial fission has long since been believed to occur at marked “fission” sites where outer mitochondrial membrane fission proteins such as MFF and Fis1 accumulate. It has recently been shown that these fission sites often occurs at contact sites between mitochondria and the endoplasmic reticulum (ER), where the ER wraps around the mitochondria and induces constriction [26]. This constriction is critical for proper DRP1 oligomerization, as the resulting DRP1 helices are much smaller than the diameter of mitochondria [14],[27]. Interestingly, the timing between DRP1’s association with a mitochondria and the subsequent fission event varies extensively [27], suggesting that the molecular machinery responsible for driving both fission and fusion events may be organized on the mitochondria well before the actual event occurs. Our data supports this finding, in that we found that in most cases mitochondrial morphology remained relatively fixed prior the identified fission or fusion event.

Several models have been performed to interrogate the dynamic nature of mitochondrial movement, fission, and fusion [20],[21]. Analyses of mitochondrial dynamics in cervical cancer cells (HeLa) and in mouse embryonic fibroblasts (MEFs) cells identified a distinct multi-patterned series of sequential fission and fusion events [21]. Our findings complement this study; in that, we used a similar biological approach to track mitochondrial fission and fusion events to identify the various morphological and positional features that impact a future fission or fusion event. The results presented by Wang *et al*. found four distinct patterns of paired fission and fusion events: 1) fusion followed by fission, 2) fission followed by fusion, 3) fusion followed by fusion, and 4) fission followed by fission. The most commonly observed pattern of paired fission and fusion events was fusion followed by a fission event and likewise, fission followed by a fusion event [21]. Considering our data, the morphology profile of a mitochondrion that has recently fused would be a combination of the profiles from the two or more mitochondria that had fused to make it. The resulting fused mitochondria would therefore have a larger perimeter, larger area and likely, smaller solidity compared to the properties of the mitochondria just prior to fusion. Based on our results from the random forest algorithm, the new geometric profile would associate with a higher propensity to undergo a subsequent fission event compared to a fusion. The same would be true for mitochondria that had recently undergone a fission event, again, making our data consistent with this study [21].

Though there have been numerous studies investigating how mitochondrial fission and fusion are affected following perturbations of their molecular regulators; however, little work has focused on dynamic nature of mitochondria at a single-organelle level. The dynamics of mitochondrial fission and fusion have been difficult to study due to limitations in imaging resolution among additional confounding factors including mitochondrial localization and motility within the cell. To ensure that we were able to confidently track single mitochondria through time, we gathered high-resolution data from defined regions of cells with fine time resolution (5 s). We chose a labeling system that marked all mitochondria within a cell so that we were able to take into consideration not only the geometric properties of the mitochondria, but also the local density of surrounding mitochondria. The significance of both positional features, minimum distance to neighboring mitochondria, and length of nearest mitochondria, supported the idea that the density of the mitochondria along with the morphology of the mitochondria plays an important role in predicting fission and fusion event. Our data would also suggest that mitochondrial fusion in particular is influenced by the density of mitochondria. Further support for the role of mitochondrial density on mitochondrial fusion comes from a recent study by Twig *et al.,* where they demonstrated that mitochondrial motility increased the likelihood for a subsequent mitochondrial fusion event, potentially by facilitating increased interactions between neighboring mitochondria [28].

Mitochondria participate in a wide variety of cellular processes beyond the traditional role of ATP generation [29]. Mitochondrial fission and fusion play important roles to maintain mitochondrial homeostasis to ensure mitochondrial function is preserved. This is a critical as mitochondrial dysfunction is linked not only to several rare inherited mitochondrial disorders, but also several age-related diseases such as neurodegenerative and cardiac disease [3],[30],[31]. Understanding the underlying mechanisms behind mitochondrial fission and fusion may therefore provide important insights into the pathology or bring to light new therapy strategies for various disease impacted by mitochondrial dysfunction.

## Materials and Methods

### Live Cell Fluorescent Microscopy of Mitochondrial Dynamics

Monoclonal populations of U2OS cells (ATCC) expressing mito_EYFP were generated following serial dilutions of U2OS cells stably expressing mito_EYFP. McCoy’s 5A media was supplemented with 500 µM G418, 48 hours following transfection, and clonal population were isolated. A medium expressing clone (U2OS_mitoEYFP) was selected for live cell analysis. U2OS_mitoEYFP cells were seeded at a density of 7.5×10^4^ cells on number 1.5 coverglass, 35 mm glass bottom culture dishes (MatTek) 72 hours prior to imaging. Mitochondrial morphology was altered through targeted knockdown of mitochondrial fusion regulator OPA1. All movies were started 48 hrs after knockdown. Live cell experiments were performed in a live cell chamber, maintaining a humid environment at 37°C and 5% CO_2_, which surrounded the microscope stage of a Nikon Ti Eclipse fluorescent microscope. Imaging of U2OS_mitoEYFP was performed in the FITC channel (25% lamp power, 100 ms exposure, ND4 and ND8 filters in the light path) using a 60x oil immersion objective. DIC images (40 ms exposure) were taken simultaneously with FITC images when the outline of the cell was required for later imaging processing and quantification. For single cell tracking, NIS Elements software (Nikon) was used to image several positions for each acquisition.

Polyclonal populations of U2OS cells expressing mito_PAGFP (U2OS_PAGFP) were generated following selection with 500 µM G418 for 72 hours following transfection. Medium expressing clones were selected for live cell analysis. U2OS_PAGFP cells were seeded at a density of 75,000 cells on umber 1.5 coverglass, 35 mm glass bottom culture dishes (MatTek) 48 hours prior to imaging. Prior to imaging, cells were treated with 15 nM Mitotracker Red CMXRos (Life Technologies) for 20 minutes. Mitotracker containing media was then aspirated and replaced with pre-warmed McCoy’s 5A +10% FBS for at least 1 hour before imaging to reduce background fluorescence. Live cell experiments were performed in a live cell chamber, maintaining a humid environment at 37°C and 5% CO_2_, which sat in the microscope stage of a Nikon A1 confocal microscope. Prior to photoactivation, a single ROI was drawn around mitochondria to be activated. Next, live cell images were captured every 10 seconds for 5 minutes (the length of time it took to capture one frame in both the FITC and TxRED channel) to track dynamics between activated mitochondria and the surrounding non-activated mitochondria.

### Image Processing and Mitochondrial Quantification

Image processing and quantification was completed with NIS Elements (Nikon). Images were deconvoluted using a 2D Fast Deconvolution function with the following settings; specimen thickness: *thick*, image noise level: *noisy*, contrast enhancement: *strong*. Following deconvolution, a top hat morphological transformation was performed by processing on intensity using a 3×3 pixel matrix (counts: 5). Regions of interest (ROI) were drawn around the mitochondrial containing area of the cell allowing for single cell analysis. The intensity profile for each ROI was imported into the statistical program, R, [32] for determination of intensity threshold limits to be used for detection and measurement of mitochondria.

Transformation (square root transformation) of the intensity profile for each ROI revealed an intensity distribution containing a consistent shoulder located on the right-hand side of the distribution (Figure 2B). Treating the distribution as one made up of two empirical distributions (labeled Distributions 1 and 2), we calculated the location of the junction between the two distributions to serve as the lower threshold limit for the image. Using R we computed the intensity value at the junction between distributions 1 and 2 by assuming a normal distribution for Distribution 1 [32]. The mean for Distribution 1 was calculated by determining the mode of the entire intensity profile. The lower threshold limit was set to the value 3 standard deviations from the mean thus excluding all pixels of intensities within the “first” distribution. Each ROI was binarized by applying a threshold that recognized all pixels of intensity above the lower threshold limit (located in Distribution 2) calculated by the ROI intensity profile (additional thresholding filters were set as follows: clean: 2x, separate: 3x). Objects selected by the threshold were quantified using an automated object count function that computed the number of objects along with measurements of length, width and circularity for objects identified.

### Determination of Fission and Fusion Events

Time series were processed to exclude mitochondria in the perinuclear region by focusing on small snapshots (22 µm×22 µm) of the periphery of five cells. The grayscale fluorescence intensity of mitochondria within these areas was thresholded to define the outline of a mitochondrion used for further analysis. Although certain fission and fusion events are evident by inspection, we designed an automatic procedure to make an unbiased determination of whether a mitochondrion underwent a fission event, fusion event, or neither. A reference image (Frame 1) was chosen and compared with the subsequent image (Frame 2) in time (i.e., the next frame in the time series of images). A relationship between a mitochondrial region in Frame 1 and a mitochondrial region in Frame 2 was recorded if at least one of two conditions was obtained: (1) the number of pixels from a region in Frame 2 contained in a region in Frame 1 was a maximum over all regions in Frame 1, or (2) the number of pixels from a region in Frame 1 contained in a region in Frame 2 was a maximum over all regions in the subsequent image. Drift was minimal and could be neglected. A score for each region *j* in Frame 2 was given by
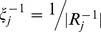
where 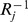 is the set of regions in the previous image having a relationship with region *j,* and 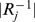 indicates the number of regions in the set. A score for each region *i* in Frame 1 was calculated using



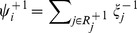



Where 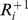 is the set of regions in Frame 2 that has a relationship with region *i.* For each region in Frame 1, if 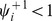, then region *i* was labeled as a region to undergo a subsequent fusion event, if 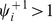, then region *i* was labeled as a region to undergo a subsequent fission event. If 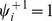, then region *i* was labeled as stable, meaning that it will undergo no fission or fusion event in the subsequent frame. As a consistency check, we performed the same computation for each region preliminarily labeled to undergo a fission or fusion event in the Frame 1 by comparing it with the image two frames (Frame 3) ahead:
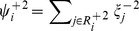



If for a given region *i* in Frame 1, both 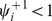 and 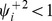, then a fusion event was determined to have taken place following the acquisition of Frame 1, and region *i* was considered to have undergone a fusion event in Frame 2. Similarly, if both 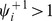 and 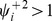, then a fission event was determined to have taken place following the acquisition of Frame 1, and region *i* was considered to have undergone a fission event in Frame 2. This calculation was repeated for each image in the dataset.

### Morphological Characterization of Mitochondria

Each region classified to undergo a fission or fusion event was scored according to six morphological features (area, extent, solidity, perimeter, eccentricity, and Euler number) using the regionprops function in MATLAB (The Mathworks, Natick, MA). Area denotes the 2D area (in µm^2^) projected to the focal plane of the image. Extent indicates the fraction of pixels in the smallest rectangle completely containing the 2D projection of the mitochondrion which are also contained in the mitochondrion itself. Solidity is a similar measure using instead the fraction of pixels in the smallest convex polygon containing the projection of the mitochondrion. Perimeter indicates the sum of edge pixels of the mitochondrial region multiplied by the length in microns of each pixel. Eccentricity is the eccentricity of the ellipse having the same second moments as the mitochondrial area. Euler number is a topological measure indicating the number of holes in the mitochondrion. We computed two additional morphological features for each region: the number of necks and the width of the smallest neck. This was accomplished by computing the pairwise distance between each pixel in the perimeter of the region, smoothing the resultant matrix with a Gaussian kernel of width 5 pixels, and finding the local minima. The number of unique pixel pairs corresponding to local minima in this matrix was the number of necks, and the value of the global minimum was the width of the smallest neck. We also computed three positional features for each mitochondrion: the distance through cytosol to its nearest neighbor, the length of surface of neighboring mitochondria within 10 µm, and the orientation of the major axis of the mitochondrion relative to the nucleus of the cell. This quantity was included based on the hypothesis that orientation of microtubules relative to mitochondrial orientation may contribute to fission propensity, where the nucleus-to-plasma-membrane radial axis was used as a proxy for microtubule orientation.

### Predictive Discrimination of Fission and Fusion Events

We used a supervised machine learning algorithm to classify the set of mitochondria undergoing fission or fusion events into two classes, based on the 11 quantitative features measured for each mitochondrial region [33]. In other words, these features were used to train a random forest [34] classifier to predict whether a mitochondrion will fuse or fragment given that one event or the other will occur in the subsequent frame (i.e., within 5 s). Using the randomforest-matlab tool [33], we “grew” 2,000 trees for each forest with the algorithmic parameter entry set to 3. In Figure 6B–E we set decision boundaries for each feature by training a single classification tree (the most basic unit of the random forest algorithm) [35] with only the data for a single morphological feature, fixed to a single bifurcation value.

### siRNA Transfection

U2OS_mitoEYFP cells were seeded into a 6 well plate at a density of 7.5×10^4^ cells per well in McCoy’s 5A (Invitrogen) supplemented with 10% FBS (MediaTech) and cultured at 37°C, 5% CO_2_ for 24 hours before transfecting with siRNA. At least two siRNA molecules against mitochondrial fusion regulator, OPA1 were obtained from Qiagen (OPA1 1: Hs_OPA1_5, OPA1 2: Hs_OPA1_9). All results were compared to control siRNA (All-star Negative Control, Qiagen). Individual siRNA sequences were transfected per well at a final concentration of 50 nM using 3 µL oligofectamine (Invitrogen) transfection reagent per well. Analysis was performed at 48 hours post knockdown as indicated in the protocol for each specific application. Knockdown of siRNA target was confirmed via western blot using primary antibodies raised against endogenous protein levels of OPA1.

### Immunoblotting

U2OS cells were lysed [in 10 mM KPO4, 1 mM EDTA, 10 mM MgCl2, 5 mM EGTA, 50 mM bis-glycerophosphate, 0.5% NP40, 0.1% Brij35, 0.1% sodium deoxycholate, 1 mM NaVO4, 5 mM NaF, 2 mM DTT and protease inhibitors (Sigma-Aldrich)] and proteins separated by SDS-PAGE. Proteins were transferred to nitrocellulose membranes and probed with an OPA1 primary antibody (BD Biosciences) overnight at 4°C followed by incubation with secondary antibodies (1 hr at room temperature, Cell Signaling). Visualization of proteins was accomplished via enhanced chemiluminescence.

### Immunofluorescence

For co-localization experiments, U2OS mito_EYFP cells were fixed in 4% methanol free formaldehyde in 1x PBS and were permeabilized with 0.2% triton x-100 in PBS. Following permeabilization, cells were blocked in blocking buffer composed of 3% bovine serum albumin (BSA) +4% goat serum in PBS. AIF, Tom20 and Alexa Fluor 555 conjugated cytochrome c, (Cell Signaling Technology – cat# 4642, BD Biosciences -cat# 612278, BD Biosciences - cat #558700, respectively) were diluted 1∶200 in blocking buffer and incubated overnight at 4°C. Alexa 555 anti-mouse or rabbit was diluted 1∶1000 in PBS for Tom20 and AIF samples respectively and incubated for 1 hour at room temperature. Nuclei were stained with Hoechst 33342 (Live Technologies) diluted in PBS to a final concentration of 2 µg/ml and incubated on cells for 15 minutes, room temperature. Images were acquired immediately afterward (mito_EYFP retention in the mitochondria diminishes overtime following fixation) with a Nikon A1 confocal microscope using a 60x oil-immersion objective (NA 1.45).

For membrane potential experiments, U2OS cells were seeded at a density of 15,000 cells per well in McCoy’s 5A media on number 1.5 coverglass in a 24 well tissue culture plate. Labeling of mitochondria with Mitotracker Red CMXros (Life technologies) was performed just prior to fixation of cells by incubating cells in 15 nM Mitotracker containing media for 20 minutes, followed by a 60 minute incubation with fresh, pre-warmed McCoy’s 5A. Cells were fixed, permeabilized, and blocked as described above. Coverglass slides were incubated in Oregon green phalloidin (Life Technologies) diluted 1∶200 in blocking buffer for 1 hour at room temperature to stain cellular actin. Nuclei were stained as described above. The coverglass pieces were inverted onto a microscope slide using Fluorogel mounting gel (Electron Microscopy Sciences) and imaged using a 60x oil-immersion objective (NA 1.4) on a Nikon Eclipse Ti fluorescent microscope.

### Seahorse Metabolic Analyzer Assays

U2OS cells passaged in McCoy’s 5A medium +10% FBS were transfected were seeded into a 6 well dish at 7.5×10^4^ cells/well. RNAi transfections were performed as described previously for 24 hours before transferring cells to a 96 well XF96 plate (Seahorse Biosciences) at a cell density of 5×10^4^ cells/well and allowed to incubate for 24 hours (experiment to begin at 48 hours post knockdown). Cartridge plates for metabolic stress injections were hydrated for at least 24 hours at 37°C with no CO_2_ prior to the assay with calibrant solution (Seahorse Biosciences).

One hour prior to running the seahorse assay, the XF96 plate’s running medium was removed and replaced with Seahorse Assay Medium (final volume 225 µl per well). Measurement of mitochondrial respiration was performed using the Seahorse XF96 analyzer under four different conditions; (1) basal, (2) 1 µM oligomycin, (3) 1 µM FCCP, (4) 1 µM rotenone and antimycin. Assay conditions and set up were performed according to instructions described by Seahorse Biosciences [36].

## Supporting Information

Figure S1 **Mito_EYFP localizes to mitochondria.** Confocal images of U2OS_mitoEYFP cells were taken following dual labeling with fluorescent antibodies against endogenous mitochondrial localized proteins; AIF, Tom20, and Cytochrome C. Pearson’s coefficient was calculated to determine the level of overlap in staining for mito_EYFP and endogenous mitochondrial markers; AIF –0.95, Tom20-0.94, and Cytochrome C –0.92.
(TIF)

Figure S2 **Loss of Membrane Potential Associates with Mitochondrial Fission.** Time-lapse images of U2OS_mitoEYGP stained with the membrane potential dependent dye, MitoTracker Red CMXros were used to track mitochondria that have lost membrane potential. The white arrows mark a mitochondrion that loses membrane potential (loss of red intensity) and undergoes subsequent mitochondrial fission.
(TIF)

Figure S3 **Confirmation of fusion events with photoactivatable GFP localized to the mitochondria.** (A) Time lapse images of U2OS stably expressing photoactivatable GFP (green) construct (PA_GFP) and stained with MitoTracker Red CMXRos (red) prior to imaging. An image of the mitochondria within the cell was captured (time = 0 sec) just before photoactivation. Mitochondria were tracked for 5 minutes (frames every ten seconds) to track in real time mitochondrial fusion events that were detected first in the red channel and later confirmed in the green. A white arrow marks a fusion event. (B) An additional example of a photoactivated fusion event.
(TIF)

Figure S4 **Mitochondrial dynamics of individual mitochondria prior to fission or fusion event.** Mitochondria identified to undergo a 1–2 fission (blue) or 2-1 fusion (red) event were tracked for 8 frames prior to the dynamic event (1 time frame −5 s) to monitor changes in mitochondrial perimeter or solidity. (A) Traces represent the tracked mean change in perimeter normalized to the perimeter of the mitochondria just prior to the fission (blue) or fusion (red) event. Shaded areas represent standard error. Normalized perimeter was calculated by determining the ratio of the perimeter of mitochondria by the perimeter of the mitochondria just prior to the dynamic event (Perimeter_0_) (B) Traces represent the tracked mean change in solidity normalized to the solidity of the mitochondria just prior to the fission (blue) or fusion (red) event. Shaded areas represent standard error. Normalized solidity was calculated by determining the ratio of the solidity of mitochondria by the solidity of the mitochondria just prior to the dynamic event (Solidity_0_).
(TIF)
