# Supplementary figures and images for: Mitochondrial Morphological Features Are Associated with Fission and Fusion Events
Source: PLoS One. 2014 Apr 14;9(4):e95265. doi: 10.1371/journal.pone.0095265 (PMC3986258; doi:10.1371/journal.pone.0095265)

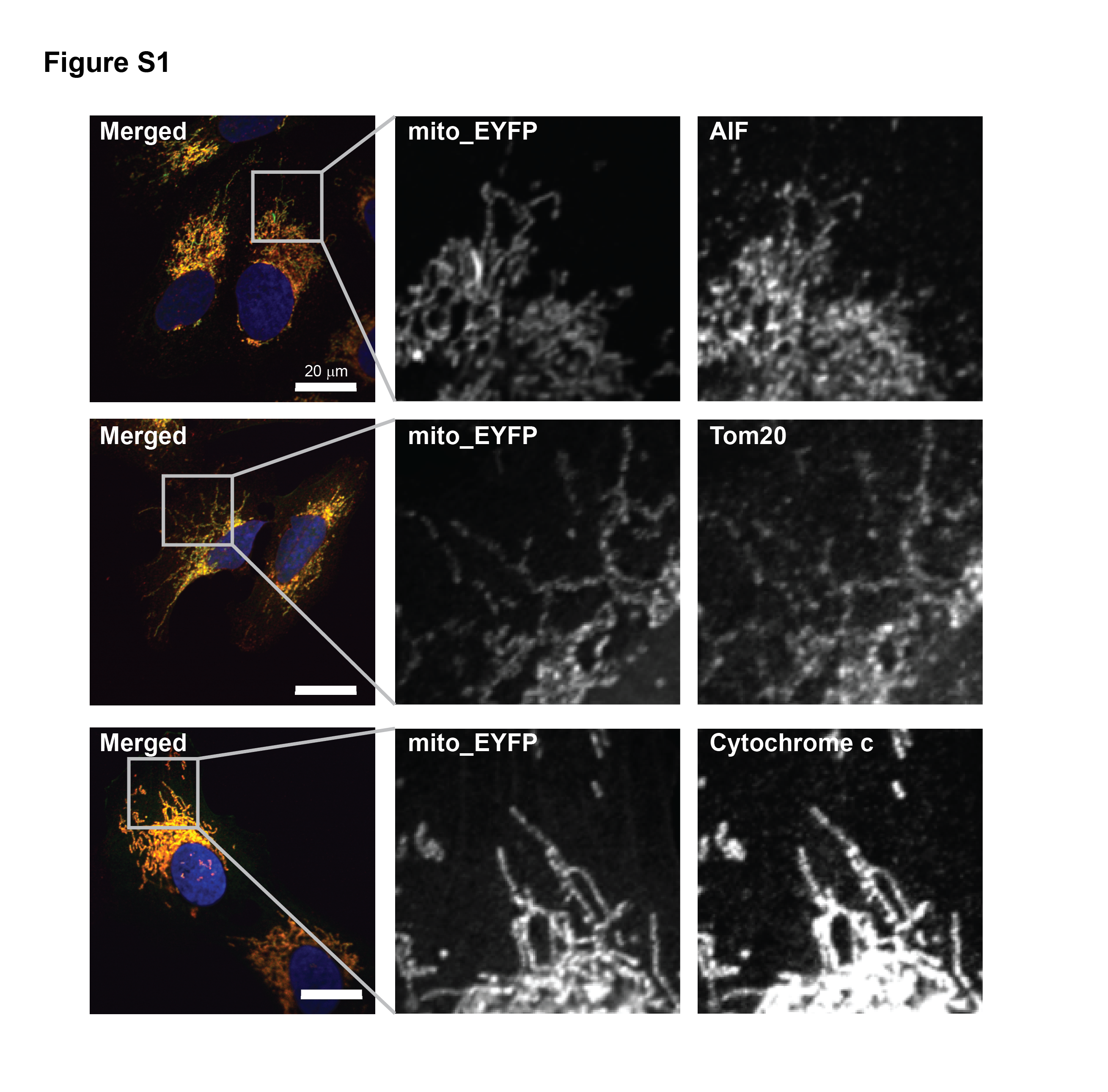

Supplement: Figure S1 — Mito_EYFP localizes to mitochondria. Confocal images of U2OS_mitoEYFP cells were taken following dual labeling with fluorescent antibodies against endogenous mitochondrial localized proteins; AIF, Tom20, and Cytochrome C. Pearson’s coefficient was calculated to determine the level of overlap in staining for mito_EYFP and endogenous mitochondrial markers; AIF –0.95, Tom20-0.94, and Cytochrome C –0.92. (TIF) [file pone.0095265.s001.tif]

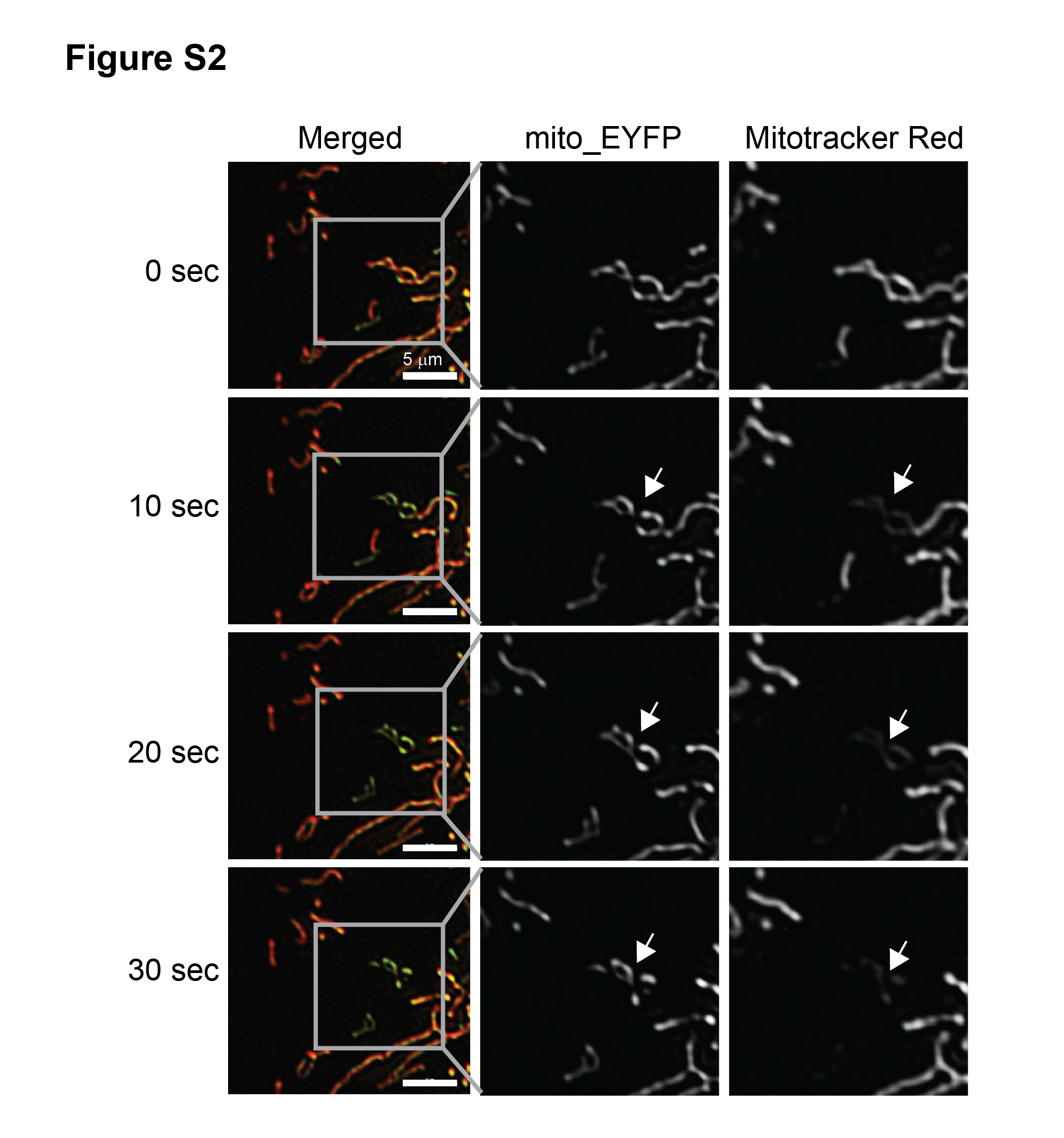

Supplement: Figure S2 — Loss of Membrane Potential Associates with Mitochondrial Fission. Time-lapse images of U2OS_mitoEYGP stained with the membrane potential dependent dye, MitoTracker Red CMXros were used to track mitochondria that have lost membrane potential. The white arrows mark a mitochondrion that loses membrane potential (loss of red intensity) and undergoes subsequent mitochondrial fission. (TIF) [file pone.0095265.s002.tif]

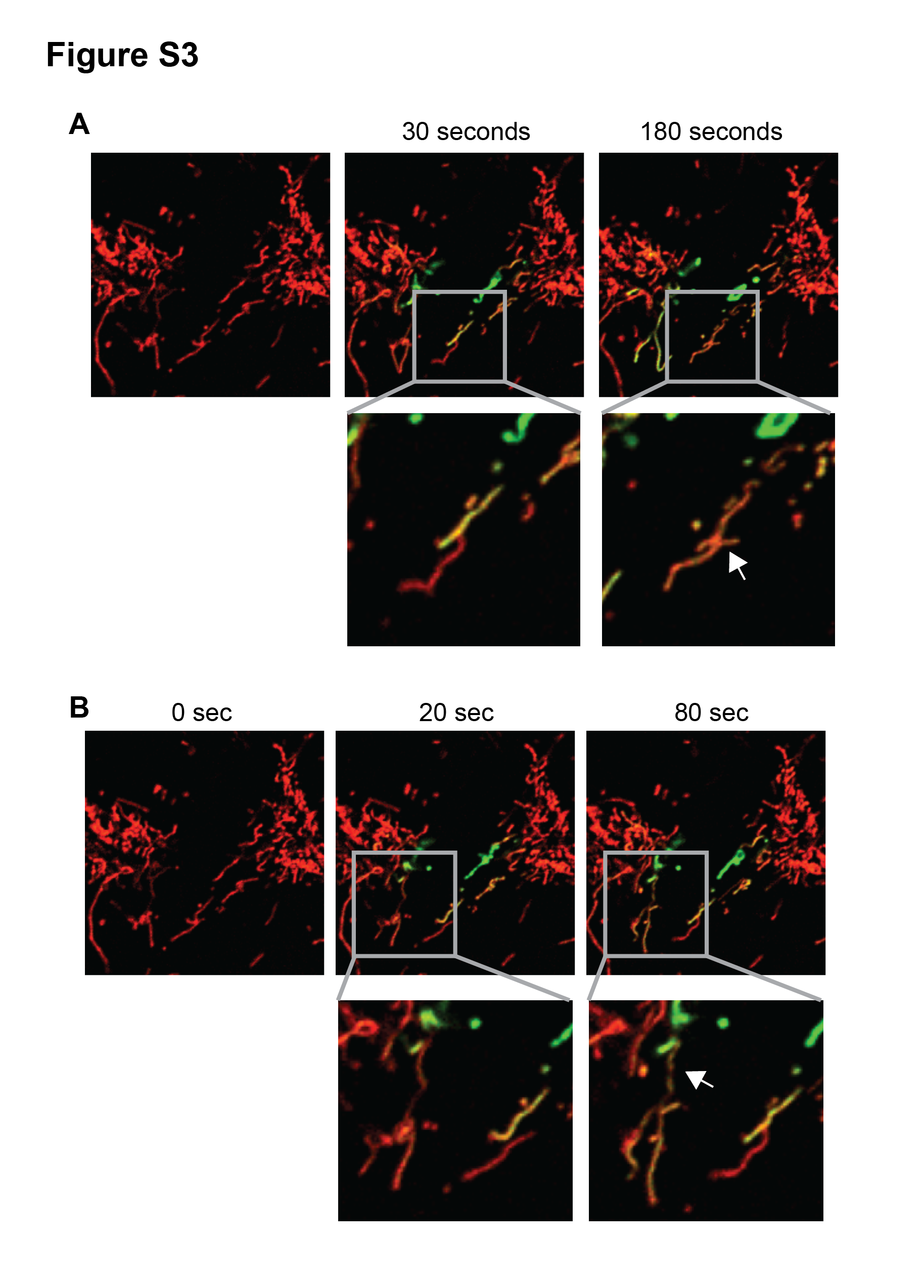

Supplement: Figure S3 — Confirmation of fusion events with photoactivatable GFP localized to the mitochondria. (A) Time lapse images of U2OS stably expressing photoactivatable GFP (green) construct (PA_GFP) and stained with MitoTracker Red CMXRos (red) prior to imaging. An image of the mitochondria within the cell was captured (time = 0 sec) just before photoactivation. Mitochondria were tracked for 5 minutes (frames every ten seconds) to track in real time mitochondrial fusion events that were detected first in the red channel and later confirmed in the green. A white arrow marks a fusion event. (B) An additional example of a photoactivated fusion event. (TIF) [file pone.0095265.s003.tif]

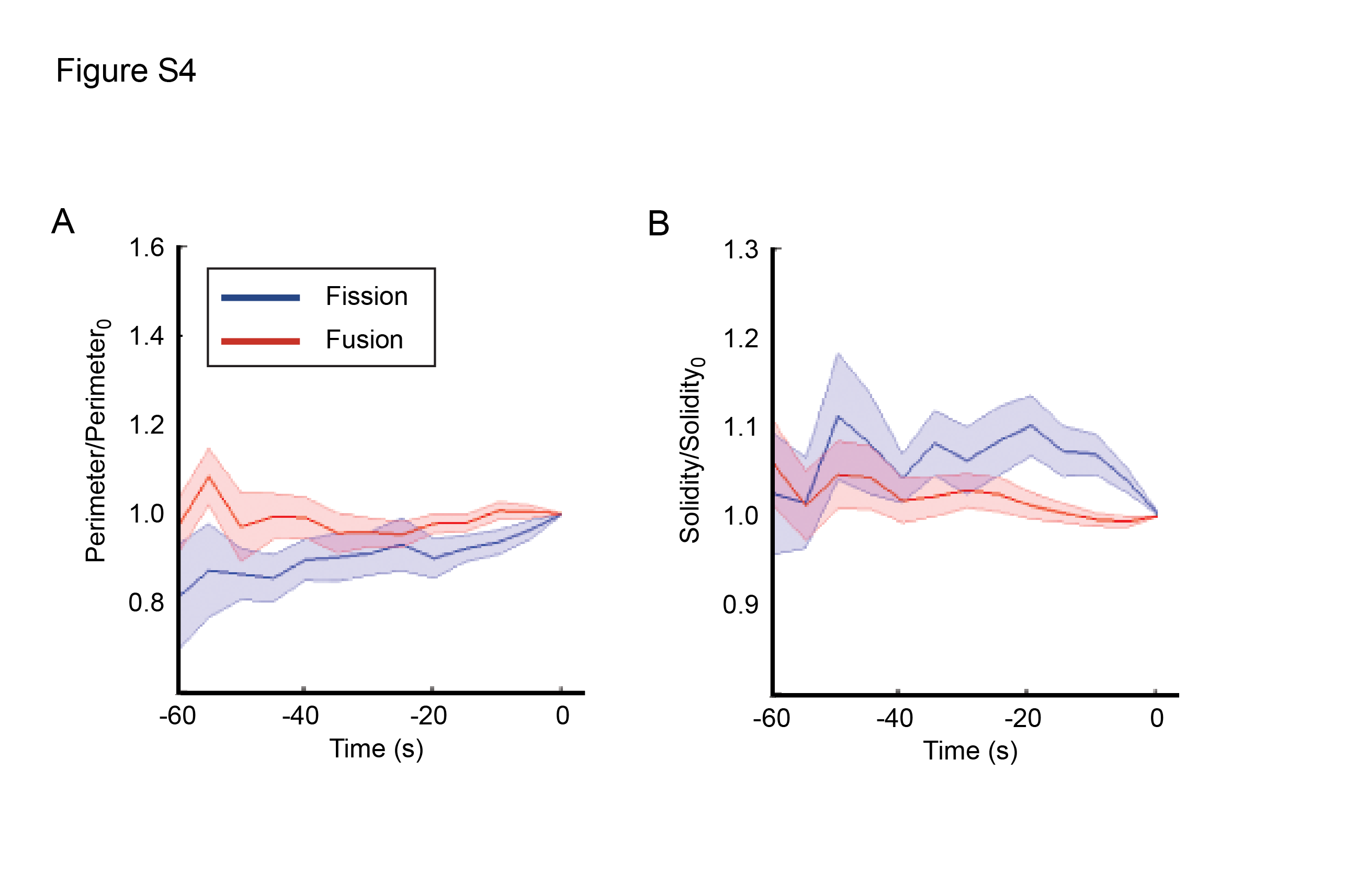

Supplement: Figure S4 — Mitochondrial dynamics of individual mitochondria prior to fission or fusion event. Mitochondria identified to undergo a 1–2 fission (blue) or 2-1 fusion (red) event were tracked for 8 frames prior to the dynamic event (1 time frame −5 s) to monitor changes in mitochondrial perimeter or solidity. (A) Traces represent the tracked mean change in perimeter normalized to the perimeter of the mitochondria just prior to the fission (blue) or fusion (red) event. Shaded areas represent standard error. Normalized perimeter was calculated by determining the ratio of the perimeter of mitochondria by the perimeter of the mitochondria just prior to the dynamic event (Perimeter0) (B) Traces represent the tracked mean change in solidity normalized to the solidity of the mitochondria just prior to the fission (blue) or fusion (red) event. Shaded areas represent standard error. Normalized solidity was calculated by determining the ratio of the solidity of mitochondria by the solidity of the mitochondria just prior to the dynamic event (Solidity0). (TIF) [file pone.0095265.s004.tif]
